# Gendered power dynamics and threats to sexual and reproductive autonomy among adolescent girls and young adult women: A cross-sectional survey in three urban settings

**DOI:** 10.1371/journal.pone.0257009

**Published:** 2021-11-29

**Authors:** Michele R. Decker, Shannon N. Wood, Meagan E. Byrne, Nathalie Yao-N’dry, Mary Thiongo, Peter Gichangi, Funmilola M. OlaOlorun, Alain K. Koffi, Scott Radloff, Saifuddin Ahmed, Amy O. Tsui

**Affiliations:** 1 Department of Population, Family and Reproductive Health, Johns Hopkins Bloomberg School of Public Health, Baltimore, Maryland, United States of America; 2 Department of Population, Family and Reproductive Health, Bill & Melinda Gates Institute for Population and Reproductive Health, Johns Hopkins Bloomberg School of Public Health, Baltimore, Maryland, United States of America; 3 Association Ivoirienne pour le Bien-Etre Familial (AIBEF), Abidjan, Côte d’Ivoire; 4 International Centre for Reproductive Health-Kenya, Nairobi, Kenya; 5 College of Medicine, University of Ibadan, Ibadan, Nigeria; 6 Department of International Health, Johns Hopkins Bloomberg School of Public Health, Baltimore, Maryland, United States of America; University of Westminster, UNITED KINGDOM

## Abstract

**Background:**

Gendered economic and social systems can enable relational power disparities for adolescent girls and young women (AGYW), and undercut autonomy to negotiate sex and contraceptive use. Less is known about their accumulation and interplay. This study characterizes relationship power imbalances (age disparity, intimate partner violence [IPV], partner-related fear, transactional sex, and transactional partnerships), and evaluates associations with modern contraceptive use, and sexual/reproductive autonomy threats (condom removal/“stealthing”, reproductive coercion, ability to refuse sex, and contraceptive confidence).

**Methods:**

Cross-sectional surveys were conducted with unmarried, currently-partnered AGYW aged 15–24 recruited via respondent-driven sampling in Abidjan, Côte d’Ivoire (n = 555; 2018–19), Nairobi, Kenya (n = 332; 2019), and Lagos, Nigeria (n = 179; 2020). Descriptive statistics, Venn diagrams, and multivariate regression models characterized relationship power imbalances, and associations with reproductive autonomy threats and contraceptive use.

**Findings:**

Relationship power imbalances were complex and concurrent. In current partnerships, partner-related fears were common (50.4%_Nairobi_; 54.5%_Abidjan;_ 55.7%_Lagos_) and physical IPV varied (14.5%_Nairobi_; 22.1%_Abidjan_; 9.6%_Lagos_). IPV was associated with reproductive coercion in Nairobi and Abidjan. Age disparate relationships undermined confidence in contraception in Nairobi. In Nairobi and Lagos, transactional sex outside the relationship was associated with condom stealthing.

**Interpretation:**

AGYW face simultaneous gendered power differentials, against the backdrop of gendered social and economic systems. Power imbalances were linked with coercive sexual/reproductive health experiences which are often underrecognized yet represent a potent link between gendered social systems and poor health. Pregnancy prevention efforts for AGYW must address reproductive autonomy threats, and the relational power imbalances and broader gendered systems that enable them.

## Background

Gender, and gender-based power disparities, are increasingly recognized as social determinants of health and wellbeing, particularly for adolescent girls and young women (AGYW) as they transition into adulthood [1]. Puberty represents an inflection point in the impact of inequitable gendered social systems, as it presents new dimensions of control, shaming and sanctioning of young women’s sexual health and burgeoning relationships, with impact at the interface of safety and sexual/reproductive health. For AGWY, gendered social and familial pressures and expectations can discourage and stigmatize contraceptive use [2–4], creating contraceptive access challenges for AGYW despite its value in reducing maternal mortality [5], enhancing child survival through birth spacing [6], and enabling agency over childbearing timing relative to education and economic goals [7, 8]. Simultaneously, adolescence represents the age of onset for intimate partner violence [9] which is responsible for over a third of women’s homicides globally [10].

Relational power imbalances at the dyad level span domains of age disparity [11, 12], economics as expressed via transactional relationships [13], and safety dynamics of violence [14] and fear [15]; all of which can compromise control over sexual and contraceptive decision-making. These dyad level experiences are enabled by gendered social and economic systems; for example, the gendered economic disparities and stratification of the workforce [1] that enable AGYWs’ economic dependence on partners, particularly older partners [12]. These experiences are often handled in silos in research and programming. Yet, domains of gendered power imbalance can interact and accumulate, for example age-disparate sexual relationships can be contexts for physical and sexual violence [11, 12] as well as financial dependence, with resulting sexual and reproductive risk. Transactional sex, i.e., that for which resources are received, can include elements of coercion [13]. A social determinants lens argues for exploration of interaction and accumulation of risk, as exemplified by the Adverse Childhood Experiences study which identified the cumulative health burden of adversity [16].

Relational threats specific to sexual/reproductive health agency can also inhibit successful contraceptive use. Leading threats to reproductive autonomy include inability to refuse unwanted sex and low confidence in contraceptive use. Direct threats to contraceptive autonomy include coercive condom negotiation, such as condom use resistance, non-consensual condom removal also known as “stealthing” [17, 18] which can prompt unprotected sex as well as fear of future negotiation [19, 20]. Reproductive coercion, i.e., partner interference with contraceptive and reproductive decisions, can include direct contraceptive sabotage and enables unintended pregnancy [21, 22]. The stigmatization of contraceptive use [2–4] can prompt some AGYW to rely on partners to obtain contraception.

AGWY in urban settings with high unmet contraception needs are a critical population for understanding the interface of gendered power disparities and their sexual/reproductive health impact. Disadvantaged urban settings can amplify risk to AGYW, and present gender-based harassment, pressure for sexual activity, and a pervasive threat of physical and sexual violence [23, 24].

This cross-sectional study: 1) describes a set of gendered relationship power imbalances (age disparity, partner violence, partner-related fear, transactional sex, and transactional partnership [Nairobi and Lagos only]) and their interplay, and 2) examines their associations with modern contraceptive use, and proximal threats to reproductive autonomy (condom removal/“stealthing”, reproductive coercion, ability to refuse sex, and confidence in contraceptive use), among adolescent girls and young women in three urban settings characterized by both high unmet contraceptive need, and social and economic gender disparities, specifically Abidjan, Côte d’Ivoire; Nairobi, Kenya; and Lagos, Nigeria.

### Settings profile: Family planning and gender indicators

Côte d’Ivoire has a high total fertility rate (5.0 for women ages 15–49 years) and adolescent birth rate (129 births/1000 adolescents aged 15–19 years) [25], coupled with low levels of modern contraceptive use (6.9% among 15-19-year-olds; 11.5% among 20-24-year-olds) [25]. Côte d’Ivoire’s UNDP Gender Inequality Index is 0.657 [26], and World Economic Forum indicators are 0.683 for Gender Earning Parity, and 0.545 for economic participation and opportunity gender gap [27]. Kenya has a total fertility rate of 3.9 for women ages 15–49 years) and adolescent birth rate (96.3 births/1000 adolescents aged 15–19 years), coupled with low levels of modern contraceptive use among young women (9.3% among 15-19-year-olds; 38.5% among 20-24-year-olds) [28]. Kenya’s Gender Inequality Index is 0.545 [2], with Gender Earning Parity and Economic Participation and Opportunity Gender Gap values of 0.680 and 0.598, respectively [27]. Nationally, Nigeria has a total fertility rate of 5.3 for women ages 15–49 years, although Lagos state has the lowest fertility rate at 3.7 for women ages 15–49 years. The adolescent birth rate is 106 births/1000 adolescents aged 15–19 years [29]. Nigeria’s Economic Participation and Opportunity Gender Gap value is 0.661 [27].

## Methods

This cross-sectional study was conducted in 2018–2020 with unmarried adolescent and young adults aged 15–24 years recruited via respondent-driven sampling (RDS). Site selection was based on unmet need related to adolescent pregnancy prevention, research capacity, and geographic diversity. In each study site, cross-sectional survey data collection followed a formative phase to inform RDS acceptability, logistics, and survey scope in accordance with best practices [30, 31]. Eligible seeds and recruits were unmarried male and female adolescents and youth aged 15–24 years with at least one year of local residence. At the Nairobi and Lagos sites, a fingerprint scanner was used to deter attempts at duplicate enrollment; confirmation of non-duplicate enrollment was an additional eligibility criterion. Consistent with RDS methods [32], seeds were purposefully selected to serve as the initial contacts for recruiting from the target population. Seeds catalyzed peer-to-peer recruitment via coupons (up to three per person) until the target sample size was achieved. Following determination of eligibility and informed consent, seed participants and subsequent recruits completed a survey. All procedures were conducted in the local language (French in Abidjan, Swahili or English in Nairobi, and Yoruba or English in Lagos). Parental consent for minors under age 18 was waived. To maximize confidentiality and accuracy, and minimize bias [33], the survey was self-administered via a handheld tablet. Staff assistance was available in cases of limited literacy or tablet unfamiliarity. All participants were provided with a local resource sheet. Procedures were approved by Institutional Review Boards at Johns Hopkins Bloomberg School of Public Health and local review boards (Comité National d’Ethique des Sciences de la Vie et de la Santé (CNESVS) of the Ministry of Health and Public Hygiene, Côte d’Ivoire; the Ethics Review Committee at Kenyatta National Hospital/University of Nairobi, Kenya; and the Health Research and Ethics Committee at the Lagos State University Teaching Hospital).

The current analysis restricts to women with previous sexual experience and current partners in Nairobi (n = 332), Abidjan (n = 555), and Lagos (n = 179).

### Measures

The full survey instrument is available at pmadata.org. Prior to implementation, the survey was tested and finalized in conjunction with the community-based research team and youth inputs.

#### Relationship power disparities across age, economic imbalance, and safety

Age disparity with current partner was defined as difference between female participant’s own age and male partner’s age of <3 years or > = 3 years.

Current transactional partnership was defined as having current partner provide any of the following: money, food, gifts, safety, shelter, transportation, or other (Nairobi and Lagos only).

Lifetime transactional sex outside relationship was assessed through a single item: “Outside of your relationship, have you ever received any of the following in exchange for sexual intercourse?” Responses comprised money, food, gifts, safety, shelter, transportation, or other, with affirmative response to any indicating transactional sex outside of partnership.

Current physical intimate partner violence (IPV) was assessed via single item “Has your partner ever pushed you, thrown something at your that could hurt you, punched you, or slapped you?”

Partner-related fear was assessed via single binary item “I try not to cause any problems with my partner because I am afraid of what he might do.”

#### Contraceptive use and reproductive autonomy threats

Modern method use: Modern contraceptive use was assessed for all sexually-active women using standard items [34]. Women were asked if they were “currently doing something or using any method to delay or avoid getting pregnant.” Method mix was then assessed among current users; modern methods comprised female sterilization, male sterilization, implant, IUD, injectables, oral contraceptive pills, emergency contraception, male condom, female condom, cycle beads, standard days, and lactational amenorrhea method.

*“Stealthing”/condom removal*. Lifetime experience of condom removal was assessed via single item: “has a partner ever agreed to use a condom and then removed it during sex?”

*Reproductive coercion*. Lifetime experience of reproductive coercion was assessed via single item “has a partner ever pressured you not to use birth control, taken your birth control (like pills) away from you, or kept you from going to the clinic to get birth control?”

*High capability of avoiding unwanted sex*. A single item, “If I do not want to have sex with my partner, I am capable of avoiding it,” enabled responses on a 4-point Likert (1-Very Capable to 4-Not Capable at All). A dichotomous variable was created to reflect high capability (score of 1) vs. all other responses.

*Confidence in using contraception*. A single item “If I want to use contraception with my partner, I can,” supported 4-point Likert responses ranging from 1-Very confident to 4-Not at all confident. Responses were dichotomized to reflect high confidence (score of 1) vs. all other responses.

*Fear-related partner procurement*. Contraceptive users indicating either entire or partial dependence on others for method obtainment were then asked a multi-select item to capture reasons for reliance. Affirmative responses to fear that method would be denied, fear of someone seeing obtain, and fear of provider shame were indicative of fear-related partner procurement; no dependence or reliance for other reasons such as convenience or partner responsibility were deemed to have no fear-related procurement.

### Analysis

All analyses were stratified by study site (Nairobi, Abidjan, and Lagos), incorporated the complex survey design, and account for clustering by node. All RDS estimates were weighted using RDS-II (Volz-Heckathorn) weights, developed using RDS-Analyst software [35], which accounts for differences in reported network size as a proxy for likelihood of receiving a coupon. To account for modest demographic differences between the RDS sample and underlying population of unmarried 15-24-year-olds in each site, a post-estimation weight was developed based on the latest national Demographic and Health Survey data (Kenya, 2014; Cote d’Ivoire 2011–12; Nigeria, 2018) and is used in conjunction with the RDS-II weight. Following diagnostics, weights were trimmed to mitigate potential biases introduced by outliers. Contention exists regarding the use of RDS survey weights for analysis; for consistency with weighted bivariate estimates presented, all models are weighted. A sensitivity analysis with unweighted models revealed modest differences in statistical significance that favored the unweighted models; weighted results are presented for the most conservative approach. All statistical testing accounted for the non-independence introduced by RDS. Analyses were conducted in Stata 16 (College Station, TX). P-values <0.05 were considered statistically significant. Analyses followed complete case methods, with denominators presented when possible. Cell sizes <10 were excluded from further analyses.

#### Characterization of interplay and accumulation of relationship power disparities

Descriptive statistics were calculated for (a) sample characteristics, (b) prevalence of each relationship agency constraint by site. Venn diagrams were constructed to visualize the accumulation; bivariate frequencies and multivariate regression models quantified the prevalence ratio for experiencing each of the imbalances as a function of the presence of a given exposure.

#### Associations with outcomes: Modern contraceptive use and proximal threats to reproductive autonomy

Prevalence estimates for modern contraceptive use and reproductive autonomy threats were calculated for the sample and by relationship power disparities, per site. Bivariate associations between each type of power disparity and each outcome were assessed via weighted Poisson regression models. Adjusted regression models included agency constraints with P<0.1 based on bivariate models.

## Results

Participants in Nairobi were slightly older and more educated than participants in Abidjan and Lagos; no participants in Nairobi had never attended schools versus 2.1% in Lagos and 9.7% in Abidjan (Table 1). Among currently-partnered women in Nairobi, transactional relationships were normative (93.2%) and a majority reported relationship age disparity (57.2%) and partner-related fears (50.7%). Transactional sex outside the relationship (22.5%) and physical IPV (14.5%) were also common. In Nairobi, 65% of currently-partnered AGYW reported modern contraceptive use. Approximately two thirds indicated high capability of avoiding unwanted sex (69.1%), and confidence in contraceptive use (65.1%). Nonetheless, adverse reproductive outcomes persisted, including lifetime reproductive coercion (19.1%), lifetime condom removal (15.6%), and fear-related partner contraceptive procurement (9.3%).

**Table 1:** Sample characteristics, relationship power disparities, and sexual/reproductive health profile of unmarried, currently-partnered AGYW by site, weighted (n = 1,066).

	Nairobi (n = 332)	Abidjan (n = 555)	Lagos (n = 179)
	n (%)	n (%)	n (%)
*Sociodemographic characteristics*			
Age (mean (sd))	20.5 (2.1)	19.8 (2.5)	19.5 (2.3)
Highest level of school attended			
None	0 (0.0)	54 (9.7)	4 (2.1)
Primary	58 (17.6)	43 (7.7)	7 (4.0)
Post-primary/Secondary	185 (55.8)	352 (63.7)	141 (79.0)
College/University	88 (13.4)	105 (18.9)	26 (14.8)
*Relationship power disparities*			
Age disparity in current partnership	190 (57.2)	394 (77.1)	128 (71.7)
Transactional partnership* (current)	309 (93.2)	--	171 (95.4)
Partner-related fear in current partnership	165 (50.4)	288 (54.5)	98 (55.7)
Physical IPV^¥^ in current partnership	48 (14.5)	122 (22.1)	17 (9.6)
Transactional sex ever^±^	75 (22.5)	93 (16.8)	73 (40.7)
*Reproductive characteristics/threats to reproductive autonomy*			
Stealthing/condom removal^±^	52 (15.6)	225 (41.9)	46 (26.7)
Reproductive coercion^±^	66 (19.8)	73 (13.6)	50 (28.2)
High capability of avoiding unwanted sex	219 (69.1)	175 (35.0)	97 (68.6)
Confidence in contraception	205 (65.1)	264 (50.7)	91 (63.6)
Fear-related contraceptive procurement^¥^	31 (9.3)	27 (4.8)	16 (9.1)
Modern contraceptive use	214 (64.5)	363 (65.4)	59 (33.2)

*Data not collected on transactional partnership in Abidjan; excluded from further analyses in Nairobi and Lagos due to normative nature.

^¥^ Excluded from additional analyses due to small sample size; Physical IPV dropped for Lagos only.

^±^ Indicates lifetime measure, not specific to current partnership.

Complete case method; sample size floats to accommodate small amounts of missing data.

In Abidjan, currently-partnered women most commonly reported age-disparate relationships (77.1%) and partner-related fears (54.5%). As with Nairobi, IPV (22.1%) and transactional sex were less frequent (16.8%). Similar to Nairobi, the majority of currently-partnered women in Lagos reported transactional partnership (95.4%), age disparity (71.7%), and partner-related fears (55.7%); however, transactional sex was more common (40.7%). In Abidjan, 65.4% of currently-partnered AGYW reported use of modern contraceptive methods. Lifetime condom removal was commonly reported (41.9%). Confidence in contraceptive use was 50.7% and capability in avoiding sex was 35.0%. Substantial barriers to contraceptive success remained, including 13.6% lifetime reproductive coercion and 4.8% fear-related partner contraceptive procurement.

**Fig 1:**
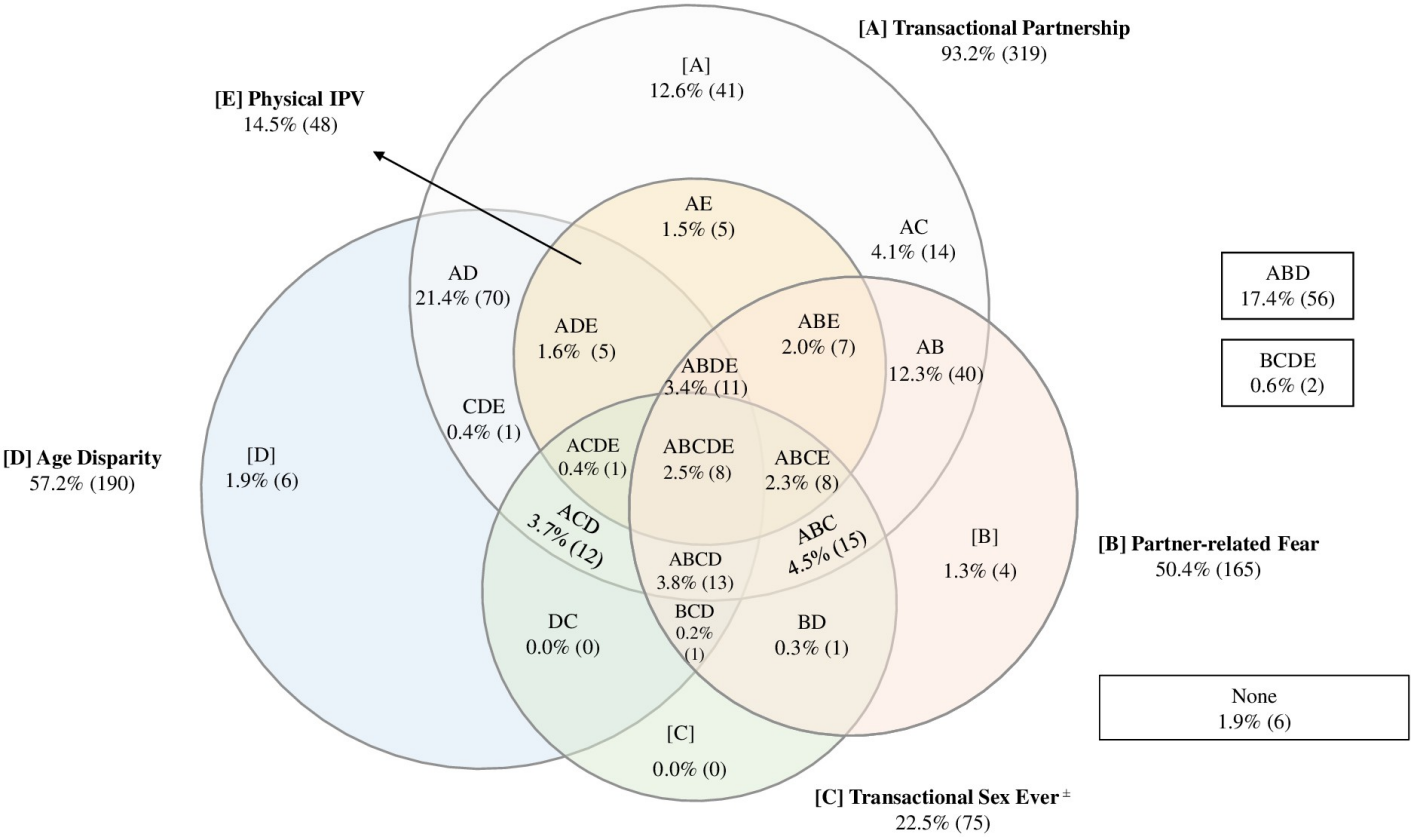
Venn diagram depicting gendered power imbalances at the interpersonal level in Nairobi, weighted (n = 332)^∝^. [A] Transactional partnership. [B] Partner-related fear. [C] Transactional sex (ever). [D] Age disparity. [E] Intimate partner violence (IPV). ^∝^cells are mutually exclusive; not proportionate to size; numbers fluctuate due to small amounts of missing data. ^±^ Indicates lifetime measure, not specific to current partnership.

**Fig 2:**
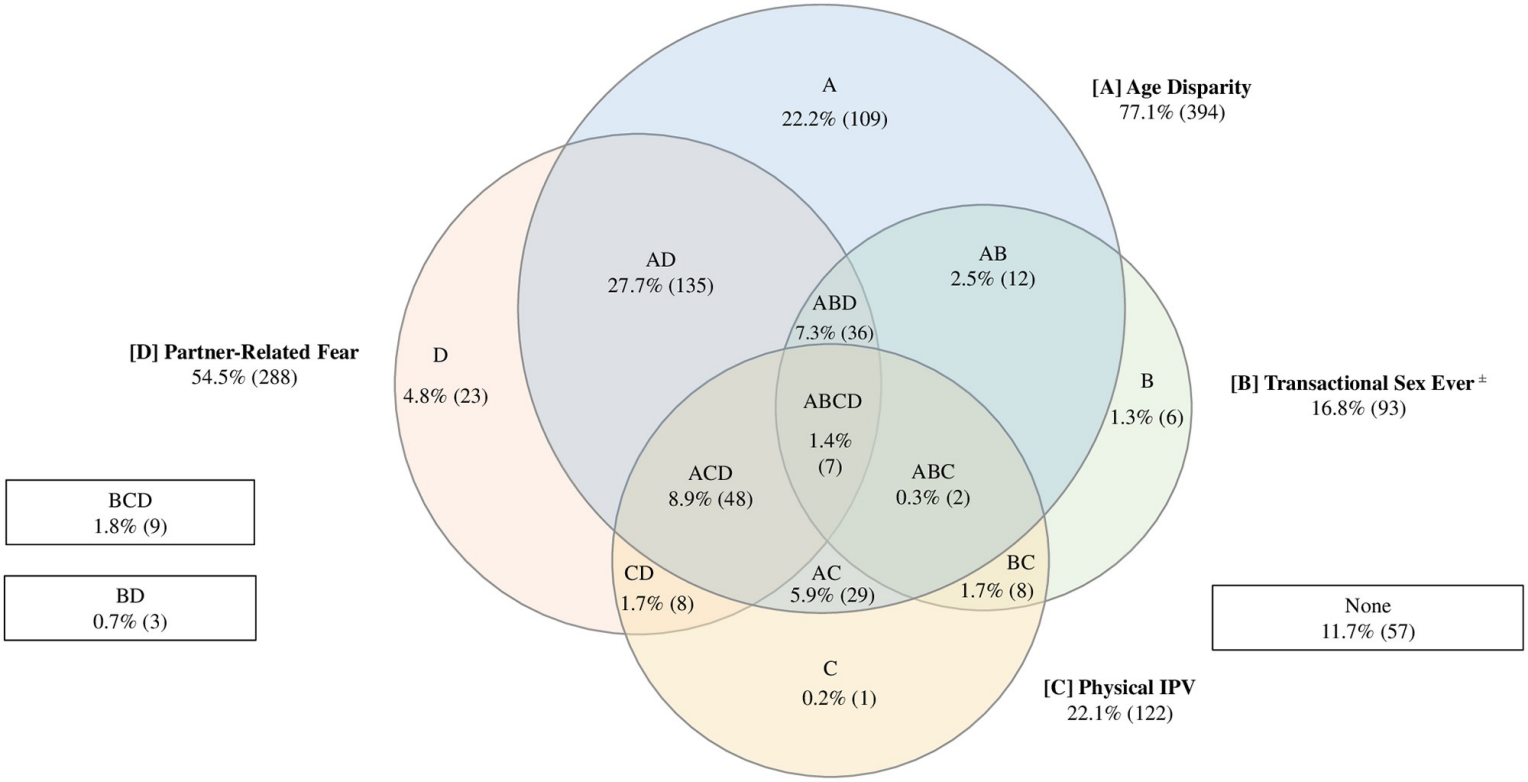
Venn diagram depicting gendered power imbalances at the interpersonal level in Abidjan, weighted (n = 555)^∝^. [A] Age disparity. [B] Transactional sex. [C] Physical Intimate Partner Violence (IPV). [D] Partner-related fear. ^∝^cells are mutually exclusive; not proportionate to size; numbers fluctuate due to small amounts of missing data. ^±^ Indicates lifetime measure, not specific to current partnership.

**Fig 3:**
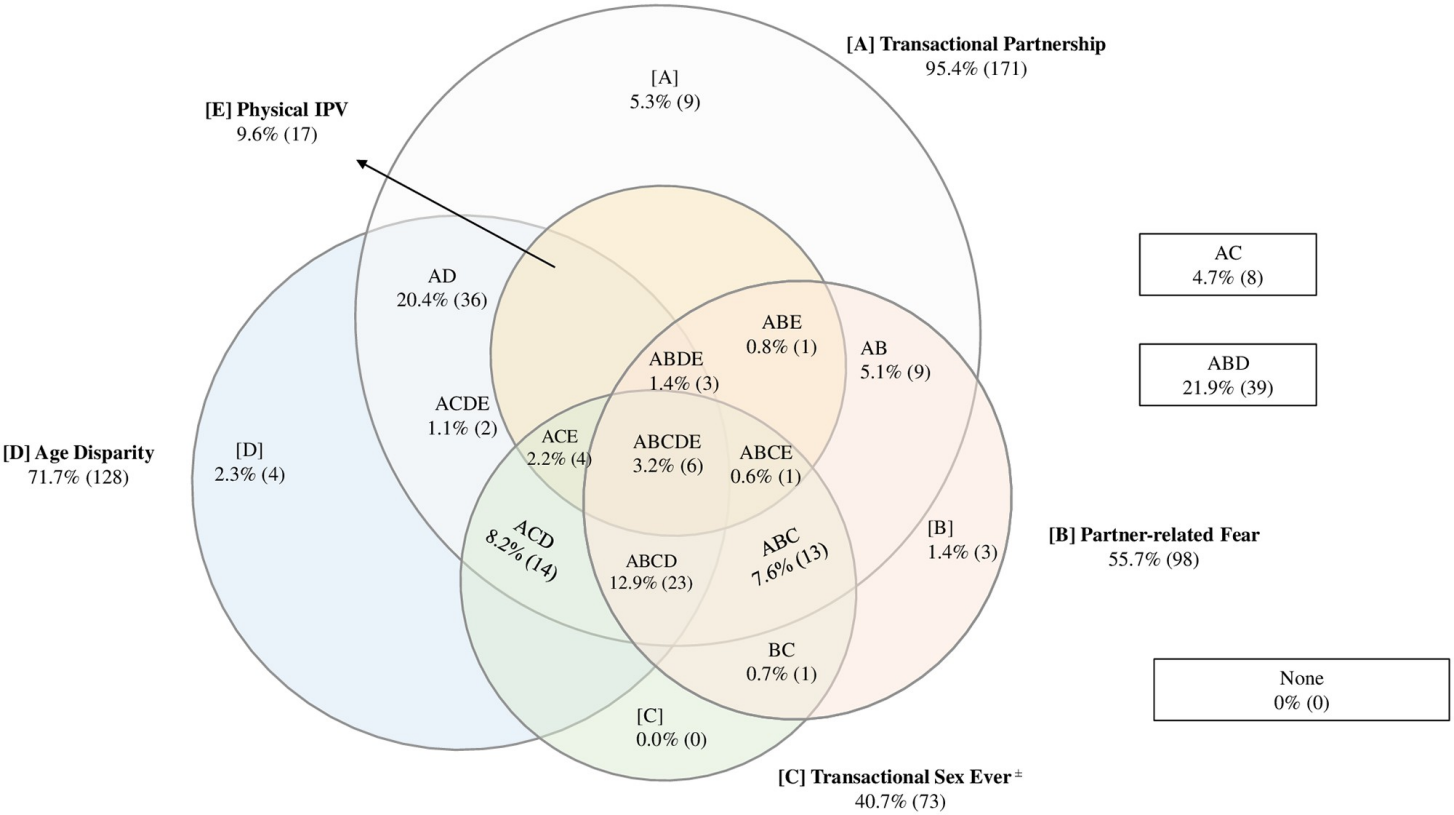
Venn diagram depicting gendered power imbalances at the interpersonal level in Lagos, weighted (n = 179)^∝^. [A] Transactional partnership. [B] Partner-related fear. [C] Transactional sex (ever). [D] Age disparity. [E] Intimate partner violence (IPV). ^∝^cells are mutually exclusive; not proportionate to size; numbers fluctuate due to small amounts of missing data. ^±^ Indicates lifetime measure, not specific to current partnership.

Patterns of relationship power disparities varied across settings (Figs 1–3). In Nairobi (Fig 1), few assessed agency constraints presented in isolation, with the exception of transactional partnerships (12.6%). The most common profiles included age disparity and transaction (21.4%), and the combination of transaction, age disparity and partner-related fear (17.4%). Similarly, in Abidjan (Fig 2), gendered interpersonal agency constraints often presented in combinations, with the exception of partner age disparity, which was reported in isolation by 22.2%. The most common combinations were age disparity in conjunction with partner-related fear (27.7%), and age disparity, partner fear and physical intimate partner violence (8.9%). In Lagos (Fig 3), agency constraints were concurrent (less than or equal to 5% in isolation). Prevalent combinations included transactional partnership, partner-related fear, and age disparity (21.9%), as well as transactional partnership and age disparity (20.4%).

When quantified with prevalence ratios, similar heterogeneity in magnitude and statistical significance was noted across settings (Table 2). In Abidjan and Lagos, transactional sex was associated with decreases in age disparate relationships (Prevalence ratio [PR]_Abidjan_: 0.34, 95% Confidence Interval [CI]: 0.12–0.94; PR_Lagos_: 0.64, 95% CI: 0.41–0.98). In Nairobi, current IPV was associated with partner-related fears (PR: 2.76, 95% CI: 1.33–5.74) and lifetime experience of transactional sex (PR: 2.51, 95% CI: 1.23–5.15). Partner-related fears (PR: 1.58, 95% CI: 1.21–2.08) and transactional sex (PR: 2.20, 95% CI: 1.24–3.92) were also associated with IPV. In Abidjan, partner-related fears were associated with age disparate relationships (PR: 1.58, 95% CI: 1.05–2.38) and lifetime experience of transactional sex (PR: 1.39, 95% CI: 1.03–1.87), and age disparate relationships further associated with partner-related fears (PR: 1.23, 95% CI: 1.03–1.49).

**Table 2:** Distribution and interplay of relationship power disparities among currently-partnered AGWY, by site, weighted.

**Nairobi (n = 332)**						
**Row %**						
	Age disparity	Partner-related fear		Physical IPV		Transactional sex^±^
Age disparity						
No		52.6		13.5		25.6
Yes		48.8		15.3		20.2
Prevalence Ratio [PR] (95% Confidence Interval [CI]I)		0.94 (0.73, 1.21)		1.06 (0.74, 1.52)		0.87 (0.62, 1.23)
Partner-related fear						
No	59.2			**7.8**		17.8
Yes	55.4			**21.4**		27.6
PR (95% CI)	0.93 (0.69, 1.24)			**1.58 (1.21, 2.08)** **		1.30 (0.97, 1.74)
Physical IPV						
No	56.7	**46.4**				**19.1**
Yes	60.1	**73.7**				**42.2**
PR (95% CI)	1.13 (0.53, 2.42)	**2.76 (1.33, 5.74)** **				**2.51 (1.23, 5.15)** **
Transactional sex^±^						
No	58.9	47.3		**10.8**		
Yes	51.4	61.2		**27.2**		
PR (95% CI)	0.79 (0.45, 1.39)	1.55 (0.91, 2.65)		**2.20 (1.24, 3.92)** **		
**Abidjan (n = 555) Row %**						
	Age disparity	Partner-related fear		Physical IPV		Transactional sex ^±^
Age disparity						
No		**37.5**		22.7		22.8
Yes		**59.4**		23.2		14.2
PR (95% CI)		**1.23 (1.03, 1.49)** *		1.01 (0.81, 1.24)		0.86 (0.64, 1.14)
Partner-related fear						
No	**67.5**			15.9		**11.4**
Yes	**83.5**			25.7		**23.0**
PR (95% CI)	**1.58 (1.05, 2.38)** *			1.28 (0.95, 1.74)		**1.39 (1.03, 1.87)** *
Physical IPV						
No	81.3	46.0				10.6
Yes	86.8	46.2				14.8
PR (95% CI)	1.34 (0.50, 3.59)	1.01 (0.36, 2.83)				1.30 (0.53, 3.19)
Transactional sex^±^						
No	**85.9**	43.2		27.9		
Yes	**62.6**	65.1		36.2		
PR (95% CI)	**0.34 (0.12, 0.94)** *	2.18 (0.70, 6.82)		1.40 (0.44, 4.44)		
**Lagos (n = 179) Row %**						
	Age disparity		Partner-related fear		Transactional sex^±^	
Age disparity						
No			57.2		55.1	
Yes			55.0		35.0	
PR (95% CI)			0.98 (0.77, 1.24)		0.79 (0.60, 1.03)	
Partner-related fear						
No	72.5				36.1	
Yes	70.7				44.7	
PR (95% CI)	0.96 (0.66, 1.40)				1.17 (0.84, 1.63)	
Transactional sex^±^						
No	**78.6**		52.0			
Yes	**61.7**		60.9			
PR (95% CI)	**0.64 (0.41, 0.98)** *		1.24 (0.77, 2.00)			

Weighted Poisson regression to estimate prevalence ratio with robust cluster seed chain = node.

*p-value significant at <0.05;

** p-value significant at <0.01.

^±^ indicates lifetime measure, not specific to current partnership.

Reproductive autonomy threats differentially impacted outcomes across contexts (Table 3). In Nairobi, AGYW in age disparate relationships > = 3 years had significantly lower confidence in using contraception (59.1%), compared to 72.9% of women with partners of similar age (Adjusted Prevalence Ratio [APR]: 0.79, 95% CI: 0.64–0.98). Physical IPV was associated with reproductive coercion (45.1% vs 15.5%, APR: 2.59, 95% CI: 1.29–4.84). For AGYW with history of transactional sex outside the relationship, lifetime stealthing/condom removal was significantly more common (29.4% vs 11.6%, APR: 2.54, 95% CI: 1.33–4.81). In Abidjan, current IPV was associated with reproductive coercion (APR: 2.31, 95% CI: 1.06–5.09). Lifetime transactional sex outside the relationship was associated with capability of avoiding unwanted sex (APR: 2.02, 95% CI: 1.35–3.02). In Lagos, transactional sex outside of the relationship was associated with stealthing/condom removal (APR = 2.27, 95% CI: 1.20–4.30) and reproductive coercion (APR: 2.62, 95% CI: 1.39–4.97).

**Table 3:** Associations between relationship power disparities, contraceptive use, and threats to reproductive autonomy among currently-partnered AGYW by site, weighted.

Outcomes (Row %)					
	Modern Contraceptive Use	Stealthing/Condom Removal^±^	Reproductive Coercion^±^	High Capability of Avoiding Unwanted Sex	Confidence in Contraception
**Nairobi (n = 332)**					
Age disparity > = 3 years					
No	67.1	14.2	20.0	73.0	**72.9**
Yes	62.6	16.6	19.7	66.1	**59.1** *
APR (95% CI)	0.93 (0.75, 1.14)	1.25 (0.64, 2.45)	1.03 (0.61, 1.75)	0.91 (0.75, 1.10)	**0.79 (0.64, 0.98)** *
Partner-related fear^‡^					
No	61.9	14.6	14.3	67.0	67.5
Yes	67.4	16.0	24.5 ^†^	70.5	63.0
APR (95% CI)	1.06 (0.85, 1.32)	0.97 (0.53, 1.78)	1.38 (0.72, 2.61)	1.05 (0.86, 1.29)	0.93 (0.75, 1.14)
Physical IPV					
No	62.6	14.6	**15.5**	70.7	67.7
Yes	76.1 ^†^	21.7	**45.1** **	59.6	49.2 ^†^
APR (95% CI)	1.22 (0.97, 1.53) ^†^	1.18 (0.55, 2.49)	**2.59 (1.29, 4.84)** **	0.84 (0.61, 1.17)	0.73 (0.51, 1.07)
Transactional sex outside relationship^±^					
No	63.9	**11.6**	17.4	70.3	64.6
Yes	66.8	**29.4** **	28.0 ^†^	64.6	66.7
APR (95% CI)	1.01 (0.80, 1.29)	**2.54 (1.33, 4.81)** **	1.27 (0.77, 2.09)	0.92 (0.71, 1.20)	1.02 (0.79, 1.31)
	Modern Contraceptive Use	Stealthing/Condom Removal^±^	Reproductive Coercion^±^	High Capability of Avoiding Unwanted Sex	Confidence in Contraception
**Abidjan (n = 555)**					
Age disparity^‡^					
No	66.7	39.3	13.7	31.8	59.0
Yes	68.1	43.0	12.5	34.9	50.4
APR (95% CI)	1.02 (0.78, 1.34)	1.09 (0.69, 1.73)	0.90 (0.34, 2.05)	1.19 (0.72, 1.97)	0.85 (0.63, 1.16)
Partner-related fear ^‡^					
No	68.2	41.9	11.5	41.4	54.5
Yes	68.9	41.6	16.4	31.5	49.4
APR (95% CI)	1.00 (0.81, 1.23)	0.99 (0.68, 1.44)	1.29 (0.55, 2.99)	0.69 (0.45, 1.05) ^†^	0.91 (0.67, 1.22)
IPV^‡^					
No	68.1	39.3	**10.7**	36.6	53.4
Yes	57.1	50.6	**24.9** *	29.4	41.3
APR (95% CI)	0.84 (0.59, 1.18)	1.29 (0.84, 1.97)	**2.31 (1.06, 5.09)** *	0.73 (0.43, 1.23)	0.77 (0.49, 1.23)
Transactional sex outside relationship^±^					
No	65.5	40.3	12.4	**29.6**	49.4
Yes	65.1	49.2	19.4	**59.7** **	56.2
APR (95% CI)	0.99 (0.75, 1.31)	1.22 (0.81, 1.84)	1.38 (0.61, 3.14)	**2.02 (1.35, 3.02)** **	1.14 (0.77, 1.67)
	Modern Contraceptive Use	Stealthing/Condom Removal^±^	Reproductive Coercion^±^	High Capability of Avoiding Unwanted Sex	Confidence in Contraception
**Lagos (n = 179)**					
Age disparity > = 3 years					
No	20.9	27.3	30.4	63.4	43.4
Yes	38.0 ^†^	26.5	27.4	69.8	68.7 ^†^
APR (95% CI)	1.82 (0.96, 3.47)	1.17 (0.60, 2.28)	1.09 (0.60, 1.97)	1.10 (0.77, 1.58)	1.50 (0.90, 2.50)
Partner-related fear^‡^					
No	34.2	20.9	**18.3**	70.6	**73.7**
Yes	31.6	32.0	**36.6** *	66.5	**54.6** *
APR (95% CI)	0.93 (0.56, 1.56)	1.44 (0.75, 2.78)	1.85 (0.94, 3.65) ^†^	0.94 (0.70, 1.26)	0.78 (0.58, 1.05) ^†^
Transactional sex outside relationship^±^					
No	37.4	**17.7**	**16.4**	70.9	60.5
Yes	27.0	**40.2** **	**45.7** **	63.9	69.4
APR (95% CI)	0.78 (0.46, 1.33)	**2.27 (1.20, 4.30)** **	**2.62 (1.39, 4.97)** **	0.90 (0.66, 1.22)	1.21 (0.91, 1.61)

Weighted Poisson regression to estimate prevalence ratio with robust cluster seed chain = node; APR adjusted all constructs significant p<0.1 in bivariate model (^†^ indicates p 0.1–0.05).

*p-value significant at <0.05;

** p-value significant at <0.01.

^‡^ complete case method; sample size floats to accommodate small amounts of missing data.

^±^ indicates lifetime measure, not specific to current partnership.

## Discussion

Complex and accumulated gender power disparities in AGYWs’ relationships were observed across three distinct urban settings. Approximately half of AGYW reported partner-related fears, and current physical IPV was present for many (14.5%_Nairobi_; 22.1%_Abidjan;_ 9.6%_Lagos_). Over half of relationships were characterized by age disparity, and transactional partnerships were so normative where assessed in Nairobi and Lagos, that the distribution precluded further analyses. Though patterns among these factors were complex and varied across settings, the assessed dimensions rarely presented in isolation, illustrating evidence of the accumulation of these power imbalances. In particular, physical IPV was virtually never observed in isolation, indicative of its embedding within a host of other power imbalances. In a time of unprecedented global recognition of gender as a social determinant of health, current results provide a much-needed evidence base for the range, nature, and health impact of relational gendered power disparities among AGYW. Relationship dyad features represent a critical means by which gendered economic and social systems influence AGYW’s safety and sexual/reproductive health. In the current era of progress towards Sustainable Development Goals 3 and 5, specific to health/contraception and gender equality, respectively, current findings advance our knowledge and ability to address power disparities at the relationship level, including those that influence contraceptive success.

The multiple simultaneous domains of relationship power disparity for AGYW demonstrated complexity and heterogeneity across settings. In Abidjan where age-disparate relationships were most prevalent at 77%, this relationship feature was significantly associated with partner-related fear. In Nairobi, partner-related fear was significantly associated with current IPV. In Nairobi, transactional sex outside the relationship was associated with current IPV; in Abidjan it was associated with partner-related fears. By contrast, in both Abidjan and Lagos transactional sex outside relationship was protective against current partnership age disparity. Transactional partnerships were normative where assessed, reflecting a confluence of gendered social and economic realities, and compromising statistical power to concurrency with other relational power dynamics and sexual/reproductive autonomy which have been linked with this experience among AGYW in other settings [36]. Consistent with best practices [37], our study differentiates exchange *partnerships* from sex trade by clarifying “within partnership” and “outside a main relationship”; though these definitions are understood as fluid. Differences in the magnitude and statistical significance of relationships assessed may reflect differences in the underlying distributions across sites, as well as heterogeneity in their impact. Further work is needed to understand relationship trajectories and the relative timing and sequencing of experiences, particularly for transactional sex outside partnerships which was assessed with a lifetime referent period.

Power imbalances were evident in sexual/reproductive health negotiations that can undermine contraceptive *success*, primarily through coercive behaviors including condom removal known as “stealthing” (15.6% _Nairobi_; 41.9%_Abidjan_; 26.7%_Lagos_), and reproductive coercion (19.8% _Nairobi_; 13.6% _Abidjan;_ 28.2%_Lagos_). The impact of these barriers to successful contraceptive use, and onward implications for unintended pregnancy are likely to be high given that male condoms are a predominant contraceptive method for this population, and coital-dependent contraceptive methods are highly susceptible to interference. In Nairobi and Lagos, transactional sex outside the partnership was associated with coercive risk in the form of stealthing history. While reproductive coercion has been previously documented in Nairobi among samples including AGYW [38]; findings provide new and valuable evidence of reproductive coercion as well as the specific behavior of stealthing among AGYW specifically in across urban sub-Saharan African settings. Consistent with evidence from other settings [39], IPV was associated with reproductive coercion in both Nairobi and Abidjan. While the timing of assessments (current IPV, lifetime reproductive coercion) does not allow conclusions about current risk, these data point to a concerning convergence of threats to both physical and reproductive safety and autonomy in the lives of AGYW.

Contraceptive confidence estimates above 50% in all three settings is promising, though current evidence of reproductive coercion and stealthing raise questions about AGWYs’ full agency over contraceptive use. Age disparate relationships undermined contraceptive confidence in Nairobi in adjusted models. Confidence in avoiding unwanted sex was lowest in Abidjan at 35%, and in the 69% range for both Nairobi and Lagos, which speaks to clear unmet needs for safety as well as autonomy in determining when, where and with whom to engage in sexual activity. In Abidjan transaction outside the partnership was associated with confidence in avoiding unwanted sex; while the relative sequencing of these experiences in unclear, results could reflect an aspect of empowerment, reflecting the diversity of exchange sex in sub-Saharan Africa [37].

Current evidence of partner violence and relationship autonomy threats is alarming yet highly actionable within the health and development sector. WHO guidelines for violence assessment and response within health settings [40] should be integrated within youth-serving clinics and can be adapted for community-based support programs focused on AGYW. Contraceptive-related discussions in both clinical settings and in sexual health promotion for lay audiences must address the risks of partner violence, reproductive coercion and stealthing [41]. Promotion of highly effective, non-coital-dependent methods can additionally serve to protect sexual and reproductive health for this population. Relationship safety assessment and planning tools have been found acceptable and valuable for safety planning among adult women [42] and may have value specific to contraceptive decision-making for AGYW.

The social determinants lens cautions against an overly individualistic response to current findings, and encourages structural change to overcome deeply entrenched gendered social and economic disparities that form the backdrop for the current results. While individual-level empowerment and safety strategies may benefit AGYW in the short term, sustainable change through structural and policy interventions to mitigate gender-based disparities, must be prioritized. The SDG framework provides important direction for progress on gender equality.

Findings should be considered in light of several limitations. Due to survey space constraints, assessments used single indicator assessments rather than fuller multidimensional assessments for key variables, including IPV, reproductive coercion, and transactional sex. The intensity and frequency of experiences were not assessed, limiting precision and ability to capture nuance and heterogeneity of experiences. The IPV measurement was limited to physical IPV and thus may underestimate prevalence. The cross-sectional nature of the study precludes inferences about temporality. Relationship dynamics were assessed for current partners however several autonomy outcomes including reproductive coercion and stealthing were assessed with a lifetime referent period, limiting precision of estimates. To avoid an assumption of exchangeability, analyses did not pursue a dose-response or scoring system.

Prevention of early and unintended pregnancy among AGYW remains a key policy priority in Kenya, Cote d’Ivoire, Nigeria and elsewhere in east and west Africa. Monitoring metrics must better integrate the gendered power imbalances, and consider threats to autonomy and contraceptive success in addition to use. The concurrence of power imbalances, while varied across sites, argues for wholistic programming that considers the interplay among related factors. Preventing unintended pregnancy through contraceptive nonuse, sabotage via reproductive coercion or condom removal, and non-volitional sex requires fostering social, economic, and interpersonal agency for young women, and shifting the social, economic, and policy systems that perpetuate these imbalances. Advancing health in this important population requires understanding and addressing gendered power imbalances at the interpersonal relationship level, within the context of broader gendered social and economic disparities.
